# Massive weight loss may facilitate otherwise impossible flap designs in phalloplasty: The first description of a tube-in-tube design of a pedicled SCIA-based flap

**DOI:** 10.1016/j.jpra.2024.11.015

**Published:** 2024-11-28

**Authors:** Wouter B. van der Sluis, Brechje L. Ronkes, Mark-Bram Bouman

**Affiliations:** a Department of Plastic, Reconstructive and Hand Surgery, Amsterdam UMC, Amsterdam, The Netherlands; b Amsterdam Public Health Research Institute, Amsterdam UMC, VU University, Amsterdam, The Netherlands; c Centre of Expertise on Gender Dysphoria, Amsterdam UMC, VU University, Amsterdam, The Netherlands; d Department of Urology, Amsterdam UMC, Amsterdam, The Netherlands

**Keywords:** Transgender, Gender dysphoria, Phalloplasty, Flaps

## Abstract

**Introduction:**

Phalloplasty with urethral lengthening (UL) is a complex procedure with a high complication rate.

**Case:**

A 44-year-old transgender man with a surgical history of mastectomy, hysterectomy, bilateral oophorectomy, colpectomy and metadoioplasty with UL wished to undergo phalloplasty with UL. He had lost 50 kgs of weight for this procedure. There was substantial skin laxity of the whole abdominal region. A tube-in-tube pedicled SCIA-based flap was designed on the left groin area. The postoperative course was complicated by a dehiscence of the urethra, for which a retubularization was performed 10 months after surgery. At 40 months of clinical follow-up, voiding while standing was possible with good urodynamic function.

**Conclusion:**

After massive weight loss, laxity of skin may facilitate otherwise impossible phalloplasty flap designs for phalloplasty.

## Introduction

Genital Gender-Affirming Surgery (gGAS) in transgender men comprises phalloplasty or metoidioplasty and can be performed with or without urethral lengthening (UL). Phalloplasty with UL is performed in transgender men who opt for surgical construction of a phallus with the desire to void in standing position. Short-term complications can include (partial) flap failure and donor-site wound healing problems, long-term complications can include neourethral strictures and/or fistulas.

A variety of flaps have been described for phalloplasty, most commonly the Radial Forearm Free Flap (RFFF), Anterolateral Thigh flap, Superficial Circumflex Iliac Artery (SCIA)-based flap, Latissimus Dorsi flap, parascapular flap or a combination of flaps.^1^ UL can be achieved by using a tube-in-tube RFFF design, labial flap, (pre)lamination with skin grafts or buccal mucosa or a combination of abovementioned flaps. A tube-in-tube design is typically described for RFFF and ALT flap phalloplasty, but not frequently for groin-based flaps.^2^^,^^3^ These individuals have to have limited subcutaneous fat, so as not to make the phallus too thick. This is the first description of a tube-in-tube design of a pedicled SCIA-based flap for phalloplasty with urethral lengthening in an individual after massive weight loss. The transgender man provided written consent for publication of this report with use of the photographs. We adhered to the STROBE guidelines.

## Case

Our institution is a tertiary referral center providing all aspects of healthcare for transgender individuals. A multidisciplinary, dedicated team of psychologists, psychiatrists, endocrinologists, plastic surgeons and urologists provides additional support for the individual in the transition process.

A 44-year-old transgender man with a surgical history of mastectomy, hysterectomy, bilateral oophorectomy, colpectomy and metadioplasty with UL wished to undergo phalloplasty with UL. In our institution, a previous colpectomy is strongly advised for individuals wanting to undergo UL, because of the lower rates of postoperative fistula.^4^

His past history included obesity and laparoscopic cholecystectomy. He met the criteria for phalloplasty, as described in the WPATH standards of care: (1) persistent, well documented gender dysphoria, (2) capacity to make a fully informed decision and to consent for treatment, (3) >18 years old, (4) no significant medical or mental health concerns, (5) 12 continuous months of hormone therapy as appropriate to the patient's gender goals and (6) 12 continuous months of living in a gender role that is congruent with their gender identity.^5^

Pre-operatively, all surgical options were discussed. He did not wish to undergo RFFF phalloplasty, because of the stigmatizing forearm scar. Furthermore, Allen's test was positive for both arms, suggesting suboptimal hand perfusion on the ulnar artery alone. He desired a SCIA-based flap because of the donor-site, that does not need skin grafting for closure. His Body Mass Index was 33 kg/m^2^, having already lost 50 kgs. At physical examination of the groin/lower abdomen, there was substantial skin laxity. Therefore, it was likely that the donor-site could be closed directly, while the girth of the neophallus would be acceptable.

**Figure 1:**
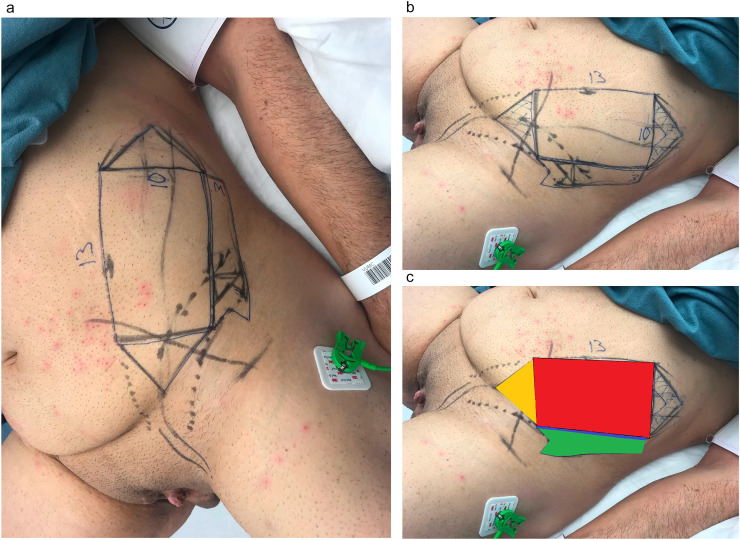
Flap design. The flap is designed on the superficial branch of the Superficial Circumflex Iliac artery. The design was comparable to the tube-in-tube design from the more commonly known tube-in-tube RFFF phalloplasty. The shaft (medial part of the flap) is rolled around the neourethra (lateral part of the flap). Figure 1c: The yellow triangle is deepithelialized, the red rectangle is used as shaft, the blue line is deepithelialized and the green part is used for neourethral reconstruction.

The SCIA-based flap is an adipofascial flap that is pedicled on the superficial branch of the SCIA. Before surgery, the superficial SCIA branch was identified using a handheld Doppler device, and marked. A pedicled flap was designed, with shaft dimensions of 13 cm long and 10 cm wide, based on the superficial SCIA branch. The design was comparable to the tube-in-tube design from the more commonly known tube-in-tube RFFF phalloplasty (Figure 1a-c). The urethral part of the flap was designed (dimensions 10 × 3 cm) lateral to the shaft, so that perforators from the deep SCIA branch could be incorporated for adequate neourethral vascularization. In other patients this design is almost always impossible, because the phallus would become too thick and/or primary donor-site closure would not be possible.

The procedure was performed under general anesthesia and lasted 281 min. Flap elevation was performed by the plastic surgery team. In this case, besides the superficial branch of the SCIA, one perforator from the deep branch was incorporated in the flap, making it not a traditional SCIP flap, but a bipedicled SCIA-based flap. A sensory nerve, probably a branch of the 12th thoracic nerve, was dissected until a length of 10 cm was achieved, and later coapted to one of the dorsal clitoral nerves. The flap was tunneled to the genital region when sufficient mobility was achieved. The donor-site was closed primarily over a suction drain.

Simultaneously, the previous metoidioplasty was used by the urology team to form the first part of the neourethra. Because of the presence of an urethral stricture in the pars fixa, dorsal inlay urethroplasty with use of local skin was performed. The remaining skin of the shaft of the metoidioplasty was mobilized leaving it vascularized with the pedicle situated on the right side. Further elongation of the neourethra was possible via this technique. After tubularization, the neourethra was anastomosed on the neourethral part of the SCIA-based flap. However, because there was too much tension on the anastomosis, an urethro-cutaneous fistula was created at the penoscrotal angle as a first stage.

Also, secondary scrotal corrections were also performed. The neourethra was anastomosed in a spatulated way. Because the phallus could not be closed tension-free, a small skin graft was used on the ventral side.

**Figure 2:**
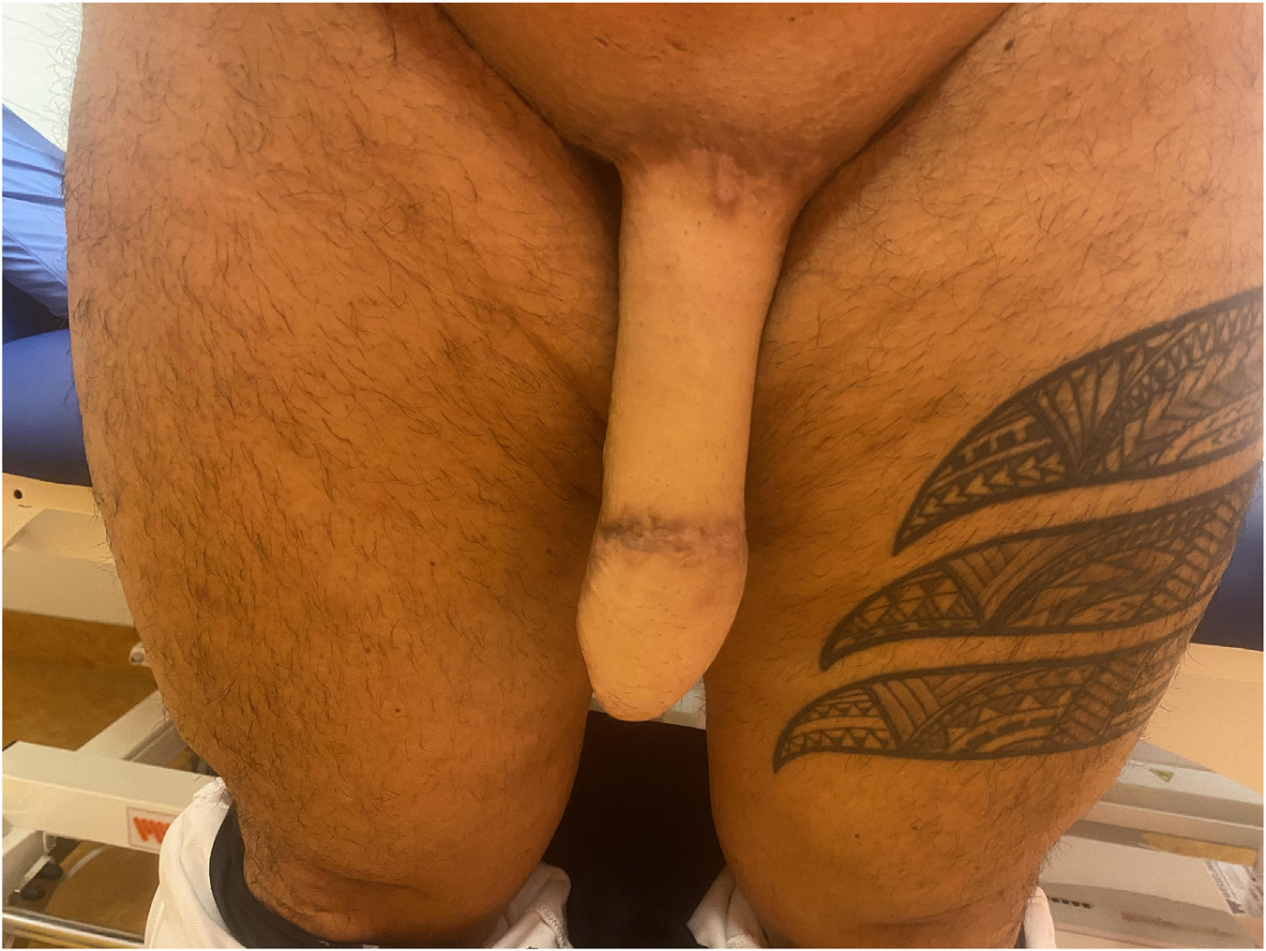
Long-term postoperative result.

The postoperative course was complicated by a dehiscence of the urethra without tissue loss, for which a retubularization was performed 10 months after surgery. Also, the patient wished a coronaplasty, which was performed at a later stage. There was no desire for erectile prosthesis implantation. At 40 months of clinical follow-up, voiding while standing was possible with good urodynamic function. The long-term postoperative result is presented in Figure 2.

## Discussion

Shared-decision making is crucial for phalloplasty flap choice. Individuals may have donor-site preferences, based on donor-site morbidity and postoperative scar size and locations. Also, ‘patient-factors’, such as the amount of subcutaneous fat, may guide the choice. Many centers only offer one or two types of flaps for the phalloplasty procedure. However, we urge gender surgeons to take into account individual factors, such as a history of massive weight loss as in this case, and look at the possibilities at an individual patient level. After massive weight loss, laxity of skin may facilitate otherwise impossible phalloplasty flap designs for phalloplasty. In this case, the design of both shaft and urethra in the flap, the ability of primary donor-site closure and acceptable girth of the phallus are unique. When using other abdominal flaps for phalloplasty, (parts of) these outcomes might be achieved by using tissue expanders and/or serial scar excision.

The neourethral dehiscence underlines how our design is “pushing it”. We were fortunate that all reconstructive goals were achieved with few reoperations and without the need for a secondary donor-site. Phalloplasty with UL has a high complication rate. Postoperative fistulas and strictures are frequent. When patient selection is optimized, the SCIA-based flap for phalloplasty has some advantages over other flaps: it is a relatively easy flap to dissect, there is no need for microvascular anastomoses and the donor-site can be closed primarily. Our patient was ideal for this type of phalloplasty, because his obesity pre-expanded the abdominal skin, while the weight loss made donor-site closure possible.

The design of the flap, in this case, caused a torsion in the neourethra. This made the urethral reconstruction more difficult and made it necessary to performed a staged construction. More experience with these kinds of flaps will confirm if this was a unique outcome this particular patient or something inherent in the design of this type of flap.

## Ethical approval

METC AUMC 2,014,312.

**Funding source:** There was no funding for this paper.

## Declaration of competing interest

None to declare.
